# The electroencephalography protocol for the Accelerating Medicines Partnership® Schizophrenia Program: Reliability and stability of measures

**DOI:** 10.1038/s41537-025-00622-0

**Published:** 2025-06-06

**Authors:** Daniel H. Mathalon, Spero Nicholas, Brian J. Roach, Tashrif Billah, Suzie Lavoie, Thomas Whitford, Holly K. Hamilton, Lauren Addamo, Andrey Anohkin, Tristan Bekinschtein, Aysenil Belger, Kate Buccilli, John Cahill, Ricardo E. Carrión, Stefano Damiani, Ilvana Dzafic, Bjørn H. Ebdrup, Igor Izyurov, Johanna Jarcho, Raoul Jenni, Anna Jo, Sarah Kerins, Clarice Lee, Elizabeth A. Martin, Rocio Mayol-Troncoso, Margaret A. Niznikiewicz, Muhammad Parvaz, Oliver Pogarell, Julio Prieto-Montalvo, Rachel Rabin, David R. Roalf, Jack Rogers, Dean F. Salisbury, Riaz Shaik, Stewart Shankman, Michael C. Stevens, Yi Nam Suen, Nicole C. Swann, Xiaochen Tang, Judy L. Thompson, Ivy Tso, Julian Wenzel, Juan Helen Zhou, Jean Addington, Luis Alameda, Celso Arango, Nicholas J. K. Breitborde, Matthew R. Broome, Kristin S. Cadenhead, Monica E. Calkins, Rolando I. Castillo-Passi, Eric Yu Hai Chen, Jimmy Choi, Philippe Conus, Cheryl M. Corcoran, Barbara A. Cornblatt, Covadonga M. Diaz-Caneja, Lauren M. Ellman, Paolo Fusar-Poli, Pablo A. Gaspar, Carla Gerber, Louise Birkedal Glenthøj, Leslie E. Horton, Christy Lai Ming Hui, Joseph Kambeitz, Lana Kambeitz-Ilankovic, Matcheri S. Keshavan, Minah Kim, Sung-Wan Kim, Nikolaos Koutsouleris, Jun Soo Kwon, Kerstin Langbein, Daniel Mamah, Vijay A. Mittal, Merete Nordentoft, Godfrey D. Pearlson, Jesus Perez, Diana O. Perkins, Albert R. Powers, Fred W. Sabb, Jason Schiffman, Jai L. Shah, Steven M. Silverstein, Stefan Smesny, William S. Stone, Gregory P. Strauss, Rachel Upthegrove, Swapna K. Verma, Jijun Wang, Daniel H. Wolf, Tianhong Zhang, Sylvain Bouix, Ofer Pasternak, Kang-Ik K. Cho, Michael J. Coleman, Dominic Dwyer, Angela Nunez, Zailyn Tamayo, Stephen J. Wood, Rene S. Kahn, John M. Kane, Patrick D. McGorry, Carrie E. Bearden, Barnaby Nelson, Scott W. Woods, Martha E. Shenton, Accelerating Medicines Partnership® Schizophrenia Program, Daniel H. Mathalon, Spero Nicholas, Brian J. Roach, Tashrif Billah, Suzie Lavoie, Thomas Whitford, Holly K. Hamilton, Lauren Addamo, Andrey Anohkin, Tristan Bekinschtein, Aysenil Belger, Kate Buccilli, John Cahill, Ricardo E. Carrión, Stefano Damiani, Ilvana Dzafic, Bjørn H. Ebdrup, Igor Izyurov, Johanna Jarcho, Raoul Jenni, Anna Jo, Sarah Kerins, Clarice Lee, Elizabeth A. Martin, Rocio Mayol-Troncoso, Margaret A. Niznikiewicz, Muhammad Parvaz, Oliver Pogarell, Julio Prieto-Montalvo, Rachel Rabin, David R. Roalf, Jack Rogers, Dean F. Salisbury, Riaz Shaik, Stewart Shankman, Michael C. Stevens, Yi Nam Suen, Nicole C. Swann, Xiaochen Tang, Judy L. Thompson, Ivy Tso, Julian Wenzel, Juan Helen Zhou, Jean Addington, Luis Alameda, Celso Arango, Nicholas J. K. Breitborde, Matthew R. Broome, Kristin S. Cadenhead, Monica E. Calkins, Rolando I. Castillo-Passi, Eric Yu Hai Chen, Jimmy Choi, Philippe Conus, Cheryl M. Corcoran, Barbara A. Cornblatt, Covadonga M. Diaz-Caneja, Lauren M. Ellman, Paolo Fusar-Poli, Pablo A. Gaspar, Carla Gerber, Louise Birkedal Glenthøj, Leslie E. Horton, Christy Lai Ming Hui, Joseph Kambeitz, Lana Kambeitz-Ilankovic, Matcheri S. Keshavan, Minah Kim, Sung-Wan Kim, Nikolaos Koutsouleris, Jun Soo Kwon, Kerstin Langbein, Daniel Mamah, Vijay A. Mittal, Merete Nordentoft, Godfrey D. Pearlson, Jesus Perez, Diana O. Perkins, Albert R. Powers, Fred W. Sabb, Jason Schiffman, Jai L. Shah, Steven M. Silverstein, Stefan Smesny, William S. Stone, Gregory P. Strauss, Rachel Upthegrove, Swapna K. Verma, Jijun Wang, Daniel H. Wolf, Tianhong Zhang, Sylvain Bouix, Ofer Pasternak, Kang-Ik K. Cho, Michael J. Coleman, Dominic Dwyer, Angela Nunez, Zailyn Tamayo, Stephen J. Wood, Rene S. Kahn, John M. Kane, Patrick D. McGorry, Carrie E. Bearden, Barnaby Nelson, Scott W. Woods, Martha E. Shenton, Gregory A. Light, Gregory A. Light

**Affiliations:** 1 Department of Psychiatry and Behavioral Sciences and Weill Institute for Neurosciences, University of California San Francisco, San Francisco, CA USA; 2 Mental Health Service, Veterans Affairs San Francisco Health Care System, San Francisco, CA USA; 3 Northern California Institute for Research and Education, San Francisco, CA USA; 4 Department of Psychiatry, Brigham and Women’s Hospital and Harvard Medical School, Boston, MA USA; 5 Orygen, Parkville, VIC Australia; 6 Centre for Youth Mental Health, The University of Melbourne, Parkville, VIC, Australia; 7 School of Psychology, University of New South Wales (UNSW), Kensington, NSW Australia; 8 University of Minnesota, Minneapolis, MN USA; 9 Washington University School of Medicine, St. Louis, MO USA; 10 Consciousness and Cognition Lab, Department of Psychology, University of Cambridge, Cambridge, UK; 11 Department of Psychiatry, University of North Carolina at Chapel Hill, Chapel Hill, NC USA; 12 UNC Intellectual and Developmental Disabilities Research Center, Carrboro, NC USA; 13 Department of Psychiatry, Yale University School of Medicine, New Haven, CT USA; 14 Department of Psychiatry, Donald and Barbara Zucker School of Medicine at Hofstra/Northwell, Hempstead, NY USA; 15 Institute of Behavioral Science, Feinstein Institutes for Medical Research, Northwell Health, Manhasset, NY USA; 16 Department of Brain and Behavioral Sciences, University of Pavia, Pavia, Italy; 17 Centre for Neuropsychiatric Schizophrenia Research, CNSR Mental Health Centre, Glostrup, Denmark; 18 Department of Clinical Medicine, University of Copenhagen, Copenhagen, Denmark; 19 Department of Psychiatry and Psychotherapy, Jena University Hospital, Jena, Germany; 20 Department of Psychology & Neuroscience, Temple University, Philadelphia, PA USA; 21 Center for Psychiatric Neuroscience, Department of Psychiatry, Lausanne University Hospital and University of Lausanne (CHUV-UNIL), Lausanne, Switzerland; 22 Department of Psychiatry, Chonnam National University Medical School, Gwangju, Korea; 23 Early Psychosis Detection and Clinical Intervention (EPIC) lab, Department of Psychosis Studies, King’s College, London, UK; 24 Department of Psychological Science, University of California, Irvine, CA USA; 25 Department of Psychiatry, IMHAY, University of Chile, Santiago, Chile; 26 Facultad de Psicología, Universidad Alberto Hurtado, Santiago, Chile; 27 Harvard Medical School, Boston, MA USA; 28 Boston VA Research Institute, Boston, MA USA; 29 Department of Psychiatry, Icahn School of Medicine at Mount Sinai, New York, NY USA; 30 Department of Neuroscience, Icahn School of Medicine at Mount Sinai, New York, NY USA; 31 Department of Psychiatry and Psychotherapy, Ludwig Maximilian University of Munich, Munich, Germany; 32 Department of Child and Adolescent Psychiatry, Institute of Psychiatry and Mental Health, Hospital General Universitario Gregorio Marañón, IiSGM, CIBERSAM, Instituto de Salud Carlos III, School of Medicine, Universidad Complutense, Madrid, Spain; 33 PEPP-Montreal, Douglas Research Centre, Montreal, QC Canada; 34 Department of Psychiatry, McGill University, Montreal, QC Canada; 35 Department of Psychiatry, Perelman School of Medicine, University of Pennsylvania, Philadelphia, PA USA; 36 Institute for Mental Health, University of Birmingham, Birmingham, UK; 37 Centre for Human Brain Health, School of Psychology, University of Birmingham, Birmingham, UK; 38 Department of Psychiatry, University of Pittsburgh School of Medicine, Pittsburgh, PA USA; 39 Department of Psychiatry and Behavioral Sciences, Northwestern University, Chicago, IL USA; 40 Olin Neuropsychiatry Research Center, Hartford HealthCare Behavioral Health Network, Hartford, CT USA; 41 School of Nursing, LKS Faculty of Medicine, University of Hong Kong, Hong Kong, China; 42 Department of Human Physiology, University of Oregon, Eugene, OR USA; 43 Shanghai Mental Health Center, Shanghai Jiaotong University School of Medicine, Shanghai, China; 44 Departments of Psychiatry and Neuroscience, University of Rochester Medical Center, Rochester, NY USA; 45 Department of Psychiatry and Behavioral Health, Ohio State University Wexner Medical Center, Columbus, OH USA; 46 Department of Psychiatry, Faculty of Medicine and University Hospital Cologne, University of Cologne, Cologne, Germany; 47 Centre for Sleep and Cognition, Yong Loo Lin School of Medicine, National University of Singapore, Singapore, Singapore; 48 Centre for Translational MR Research, Yong Loo Lin School of Medicine, National University of Singapore, Singapore, Singapore; 49 Department of Psychiatry, Hotchkiss Brain Institute, University of Calgary, Calgary, AB Canada; 50 General Psychiatry Service, Treatment and Early Intervention in Psychosis Program (TIPP–Lausanne), Lausanne University Hospital and University of Lausanne, Lausanne, Switzerland; 51 Department of Psychosis Studies, King’s College, London, UK; 52 Early Intervention for Psychosis Services, Birmingham Women’s and Children’s NHS Foundation Trust, Birmingham, UK; 53 Department of Psychiatry, University of California, San Diego, CA USA; 54 Department of Neurology and Psychiatry, Clínica Alemana—Universidad del Desarrollo, Santiago, Chile; 55 Department of Psychiatry, School of Clinical Medicine, LKF Faculty of Medicine, University of Hong Kong, Hong Kong, China; 56 Prevention Science Institute, University of Oregon, Eugene, OR USA; 57 Oregon Research Institute, Springfield, OR USA; 58 Copenhagen Research Centre for Mental Health, Mental Health Copenhagen, University of Copenhagen, Copenhagen, Denmark; 59 Department of Psychology, University of Copenhagen, Copenhagen, Denmark; 60 Department of Psychiatry, Beth Israel Deaconess Medical Center and Harvard Medical School, Boston, MA USA; 61 Department of Neuropsychiatry, Seoul National University Hospital, Seoul, Korea; 62 Department of Psychiatry, Seoul National University College of Medicine, Seoul, Korea; 63 Mindlink, Gwangju Bukgu Mental Health Center, Gwangju, Korea; 64 Department of Psychiatry, Hanyang University Hospital, Seoul, South Korea; 65 Department of Psychiatry, Washington University Medical School, St. Louis, MO USA; 66 Department of Psychology, Northwestern University, Evanston, IL USA; 67 Olin Neuropsychiatry Research Center, Hartford Hospital, Hartford, CT USA; 68 CAMEO, Early Intervention in Psychosis Service, Cambridgeshire and Peterborough NHS Foundation Trust, Cambridge, UK; 69 Institute of Biomedical Research (IBSAL), Department of Medicine, Universidad de Salamanca, Salamanca, Spain; 70 Connecticut Mental Health Center, New Haven, CT USA; 71 Douglas Research Centre, Montreal, QC Canada; 72 Department of Psychology, University of Georgia, Athens, GA USA; 73 Department of Psychiatry, University of Oxford, Oxford, UK; 74 Institute of Mental Health, Singapore, Singapore; 75 Duke-NUS Medical School, Singapore, Singapore; 76 Department of Software Engineering and Information Technology, École de technologie supérieure, Montreal, QC Canada; 77 Department of Psychiatry, Massachusetts General Hospital and Harvard Medical School, Boston, MA USA; 78 Departments of Psychiatry and Biobehavioral Sciences & Psychology, Semel Institute for Neuroscience and Human Behavior, University of California, Los Angeles, CA USA; 79 Department of Radiology, Brigham and Women’s Hospital, Boston, MA USA; 80 Veterans Affairs San Diego Health Care System, San Diego, CA USA

**Keywords:** Psychosis, Biomarkers

## Abstract

Individuals at clinical high risk for psychosis (CHR) have variable clinical outcomes and low conversion rates, limiting development of novel and personalized treatments. Moreover, given risks of antipsychotic drugs, safer effective medications for CHR individuals are needed. The Accelerating Medicines Partnership® Schizophrenia (AMP® SCZ) Program was launched to address this need. Based on past CHR and schizophrenia studies, AMP SCZ assessed electroencephalography (EEG)-based event-related potential (ERP), event-related oscillation (ERO), and resting EEG power spectral density (PSD) measures, including mismatch negativity (MMN), auditory and visual P300 to target (P3b) and novel (P3a) stimuli, 40-Hz auditory steady state response, and resting EEG PSD for traditional frequency bands (eyes open/closed). Here, in an interim analysis of AMP SCZ EEG measures, we assess test-retest reliability and stability over sessions (baseline, month-2 follow-up) in CHR (n = 654) and community control (CON; n = 87) participants. Reliability was calculated as Generalizability (G)-coefficients, and changes over session were assessed with paired t-tests. G-coefficients were generally good to excellent in both groups (CHR: mean = 0.72, range = 0.49–0.85; CON: mean = 0.71, range = 0.44–0.89). Measure magnitudes significantly (p < 0.001) decreased over session (MMN, auditory and visual target P3b, visual novel P3a, 40-Hz ASSR) and/or over runs within sessions (MMN, auditory/visual novel P3a and target P3b), consistent with habituation effects. Despite these small systematic habituation effects, test-retest reliabilities of the AMP SCZ EEG-based measures are sufficiently strong to support their use in CHR studies as potential predictors of clinical outcomes, markers of illness progression, and/or target engagement or secondary outcome measures in controlled clinical trials. Watch Dr Daniel H. Mathalon discuss their work and this article: https://vimeo.com/1066564687.

## Introduction

Estimates of the rate of conversion to psychosis in individuals at clinical high risk (CHR) followed for 2–3 years in prospective longitudinal studies have declined relative to the 40–50% rates reported in initial studies^1–5^. A recent meta-analytic review estimates psychosis conversion rates of 19–25% over a follow-up period of 2–3 years, with subsequent conversion rates decelerating over time^6^. In addition, estimates from recent large scale CHR studies are closer to 15–20%^7–10^, underscoring that only a minority of CHR individuals are likely to convert to psychosis. Moreover, CHR non-converters are clinically heterogeneous, with 20-30% remitting from the CHR syndrome over similar follow-up periods and another 30-40% remaining with persistent CHR symptoms^11,12^. These variable clinical outcomes and low conversion rates constrain our ability to develop novel treatments for the CHR syndrome, particularly if the primary target is prevention of psychosis. Further, without tools for predicting the relative likelihood of these various outcomes, our ability to personalize or stage treatments based on a CHR individual’s specific risk is limited. Accordingly, a major focus of CHR research has been to identify measures that improve the accuracy with which clinical outcomes can be predicted.

To date, several clinical risk calculators have been developed that estimate risk for conversion to psychosis using clinical, cognitive, and demographic variables^13–20^. A wide range of biomarkers have also been shown to predict outcomes in CHR individuals with modest to moderate effect sizes, suggesting that their incorporation into multivariate prediction algorithms may improve clinical prediction accuracy while also elucidating possible pathophysiological mechanisms. Improved prediction of clinical outcomes facilitates the development of new treatments, including novel drugs, informed by the CHR individual’s level of risk and likely clinical trajectory. This is the overarching framework guiding the Accelerating Medicines Partnership® Schizophrenia (AMP SCZ) Program and its assessment of multiple biomarker domains.

### Accelerating Medicines Partnership® Schizophrenia Program

The AMP SCZ program (https://www.ampscz.org/) is the largest prospective multi-site longitudinal study of CHR individuals undertaken to date worldwide^21–23^. It comprises two data collection networks, ProNET (Psychosis Risk Outcomes Network) and PRESCIENT (Prediction Scientific Global Consortium), spanning 43 sites across five continents. It is also supported by the centralized Psychosis Risk Evaluation, Data Integration, and Computational Technologies - Data Processing, Analysis, and Coordinating Center (PREDICT-DPACC).

Building on prior CHR studies, AMP SCZ aims to develop tools for predicting conversion to psychosis and other clinical outcomes in CHR individuals. The project adopts a biomarker-informed framework, incorporating both previously identified and novel measures across several clinical, cognitive, behavioral, and biological domains. Biomarkers can enhance clinical trial design by providing baseline measures for screening in/out specific patient subgroups and by serving as measures of target engagement and treatment response. This sets the stage for more rapid and efficient testing of new treatments targeting CHR symptoms and prevention of psychosis.

Finally, a major deliverable of AMP SCZ is creation of a data repository accessible to the broader scientific community through the National Institute of Mental Health (NIMH) Data Archive(NDA), providing a resource for efficient hypothesis testing, predictive tool development and validation, and other scientific discoveries (see also Billah et al.^24^ in this issue for description of AMP SCZ data flow pipeline). The software used to create the data repository is also publicly available^25,26^.

### Candidate biomarker considerations and EEG/ERP measures

According to the FDA, a biomarker is “a defined characteristic that is measured as an indicator of normal biological processes, pathogenic processes, or responses to an exposure or intervention, including therapeutic interventions” (https://www.fda.gov/drugs/biomarker-qualification-program). Because many EEG and EEG-based event-related potential (ERP) and event-related oscillation (ERO) measures serve as sensitive indices of brain function, they are often studied as potential biomarkers for various purposes in psychiatric disorders. These purposes include the prediction of illness onset or clinical outcomes, prediction of treatment response, indication of target engagement or mediation of clinical response in treatment studies, and/or tracking of illness progression^27,28^. EEG-based biomarkers are also attractive because EEG is relatively inexpensive and scalable across clinical settings^29^. Given that oscillatory frequencies present in human EEG have been conserved across mammalian evolution^30^, many EEG-based measures are also translatable to animal models^28^, thereby facilitating discovery of pathophysiological mechanisms and novel treatments that target them^27^. As noted in a recent “umbrella review” of prior systematic reviews and meta-analyses^31^, biomarker research in psychosis has been hampered by underpowered studies, a limitation that the current AMP SCZ study is poised to overcome.

During planning of AMP SCZ, EEG/ERP researchers from ProNET, PRESCIENT, PREDICT-DPACC, NIMH, Foundation for the NIH (FNIH), and pharmaceutical industry partners convened several meetings to review candidate EEG-based biomarkers for inclusion in the study. The goal was to converge on a battery of well-established measures with the greatest potential to serve as biomarkers of psychosis risk. Priority was given to EEG-based measures previously shown to predict CHR outcomes, particularly conversion to psychosis^32,33^ or remission from the CHR syndrome^34^. Priority was also given to EEG-based measures known to be abnormal in patients with schizophrenia^35–37^ or their first-degree relatives^37,38^, which was typically the case for EEG-based measures included in prior CHR studies^32^. In addition, EEG-based measures with established sensitivity to neurotransmitter/neuroreceptor mechanisms, demonstrated through pharmacological challenge studies in both humans and in animal models, were prioritized because of their potential to elucidate pathophysiology and to serve as measures of target engagement in studies of novel treatments^27,28,37^. Also prioritized were measures with prior evidence of at least moderate reliability^39^. Finally, decisions were constrained by the need to keep EEG recording time to under one hour to promote tolerance of the procedure by participants, who included symptomatic CHR individuals and children as young as age 12. Based on these considerations, the EEG team converged on a set of paradigms and measures, including mismatch negativity (MMN), auditory and visual P300, 40-Hz auditory steady state response (ASSR), and resting EEG.

#### Mismatch negativity (MMN)

MMN is an ERP component elicited by infrequent auditory “deviant” tones interspersed among frequent “standard” tones^40–42^. MMN is pre-attentive (i.e., elicited while sounds are ignored)^41,43^, N-methyl-D-aspartate (NMDA) receptor-dependent^44,45^, a reflection of auditory echoic memory^46^, and, from a predictive coding framework, a prediction error signal^47,48^. MMN amplitude deficits are well-replicated in schizophrenia and CHR studies^32,34,49,50^, including studies showing intact MMN to predict CHR remission^51,52^, and prior work showing MMN deficits to predict conversion to psychosis^32,53,54^, especially when using combined pitch+duration “double deviants”^55,56^. MMN deficits also correlate cross-sectionally with poorer functioning in CHR individuals^57^, similar to findings in schizophrenia^58,59^. Because paradigms that elicit smaller MMNs are less sensitive to schizophrenia^49,60–62^, and both pitch- and duration-deviant MMNs are reduced in schizophrenia to variable degrees across patients and studies^49,50^, the double-deviant MMN was previously implemented to maximize both MMN amplitude^63–66^ and its sensitivity to theorized heterogeneous MMN deficits across CHR individuals^55,56,67^. Accordingly, a double-deviant MMN paradigm was included.

#### Auditory and visual P300

P300 is an ERP component elicited by infrequent targets or salient distractors interspersed among frequent standards in “oddball” target detection tasks^68^. The P300 has two subtypes^68^: P3b reflects effortful “top-down” attentional shifts to target stimuli that require a response; P3a reflects automatic “bottom-up” orienting of attention to novel or salient distractors. P300 is mediated by glutamatergic transmission at NMDA receptors^69–73^ as well as by dopaminergic, noradrenergic, cholinergic, and GABAergic activity^70,74–76^. Auditory, and to a lesser degree, visual, P3b and P3a amplitude reductions have been widely replicated in schizophrenia^77,78^. In CHR studies, we and others found reduced auditory^79–81^ and visual^80^ target P3b, and less consistently, reduced auditory^82^ and visual^80^ novel P3a, to predict conversion to psychosis. Intact auditory target P3b^79^ and novel P3a^82^ have also been shown to predict remission from the CHR syndrome. Thus, both auditory and visual 3-stimulus oddball paradigms were included.

#### Gamma 40-Hz ASSR

Gamma band (30–80 Hz) neural oscillations^83,84^ arise from recurrent glutamate-mediated excitation of NMDA receptors on parvalbumin-expressing fast-spiking interneurons that subsequently release GABA, transiently inhibiting excitatory neuron firing and glutamate release^85–88^. NMDA and/or GABA receptor abnormalities are thought to underlie gamma power and phase synchrony deficits in schizophrenia^89–93^. Evidence for NMDA receptor modulation of gamma oscillations includes pharmacological challenge studies with NMDA antagonists in both humans^94–96^ and animal models^91,93,95,97–99^.

While the EEG gamma band range encompasses 30–80 Hz, 40 Hz is considered a “resonant” frequency in the auditory system because EEG power evoked by repeated auditory stimulation is largest when the driving frequency is 40-Hz^100^. Thus, the 40-Hz auditory steady state response (ASSR), typically elicited by 40-Hz click trains, has often been used in past research to assess the integrity of gamma oscillations and has been the major source of in vivo human data implicating deficient gamma oscillations in schizophrenia^101–105^. Prior studies have shown 40-Hz ASSR power and phase synchrony measures to have good test-retest reliability in both schizophrenia patients and healthy controls^106^.

The 40-Hz ASSR has been examined in several CHR studies. In the North American Prodrome Longitudinal Study-2, CHR individuals showed deficits in 40-Hz ASSR phase synchrony (i.e., inter-trial phase coherence; ITC), but not total power, between 300 and 400 ms following click-train onset relative to community controls^107^. Another study found reduced gamma ASSR ITC and total power between 300 and 500 ms in CHR individuals, relative to controls^108^. In addition, one study found that reduced 40-Hz ASSR ITC predicted conversion to psychosis in CHR individuals^109^. Thus, based on prior studies and interest in its underlying neural mechanism, the 40-Hz ASSR paradigm was included.

#### Resting EEG

Resting EEG power spectral density abnormalities are present in schizophrenia, including increased delta and theta, decreased alpha^83,110^ and increased gamma^99,111^. CHR studies have also reported that spectral EEG abnormalities predict conversion to psychosis, including increased theta and delta power, either alone^112^ (but see ref.^113^) or combined with symptom severity^114^, and decreased alpha peak frequency^112^ (but see ref.^113^). Accordingly, resting EEG was included.

### Test-retest reliability and stability of EEG measures

The AMP SCZ study assesses all biomarkers, including EEG, at baseline and at 2-month follow-up in order to examine biomarker change trajectories over a relatively short interval as potential predictors of CHR clinical outcomes. Intervals longer than 2 months were not considered because AMP SCZ emphasized predictive biomarkers with potential use in future CHR clinical trials as a means to enrich the CHR sample with those at greatest risk for converting to psychosis. A subgroup of community control (CON) participants was similarly tested at baseline and 2-months. This provided an opportunity to assess test-retest reliability of the EEG-based measures. Using G-coefficients, a type of intraclass correlation coefficient (ICC), the resulting reliability (or “generalizability”) estimates can be considered conservative estimates of the true reliabilities of the measures. This is because true systematic change, and not just random measurement error, can occur over a 2-month interval, thereby attenuating the resulting G-coefficient relative to the value expected when using a short test-retest interval during which little systematic change is expected.

For EEG candidate biomarkers to be useful as predictors of clinical outcomes, markers of disease progression, or measures of target engagement and/or treatment effects in clinical trials, they must have sufficiently high test-retest reliability. This is because reliability places an upper bound on the validity achievable by a biomarker, i.e., a biomarker cannot be expected to correlate more highly with external validation measures than it does with itself over a short test-retest interval. Accordingly, we present test-retest EEG data from an interim sub-sample of CHR and CON participants, focusing on paradigm descriptions and processed data results, as well as test-retest reliability and stability of measures, within each group. Group differences, which are not tested here, will be addressed in a future report when participant recruitment is complete.

## Methods

### Participants

To perform an interim analysis of AMP SCZ EEG measures to assess their test-retest reliability/stability, a subset of CHR (n = 654) and community control (CON; n = 87) participants who had completed baseline and 2-month follow-up EEG assessments were identified from across AMP SCZ sites. CHR participants met criteria for the CHR syndrome based on the PSYCHS (Positive SYmptoms and Diagnostic Criteria for the CAARMS Harmonized with the SIPS) structured interview^115^ and consensus review by AMP SCZ clinical experts who reviewed all CHR assessments. CON participants were screened with the PSYCHS and did not meet criteria for any psychotic disorder. For a detailed description of the AMP SCZ study design, clinical assessments, and inclusion and exclusion criteria, see ref.^21^. Each adult participant provided written informed consent, whereas minor participants provided oral assent and written parental consent. The project was approved by the governing institutional review board at each site and is registered on clinicaltrials.gov (NCT05905003).

### EEG acquisition technical challenges and choices

Here, we describe the considerations, technical challenges, and solutions adopted by AMP SCZ to optimize high quality EEG data acquisition harmonized across academic sites, continents, and languages, including site set-up, staff training and certification procedures, and the development of automated processing pipelines and procedures for data uploads, visualization, and quality control monitoring.

#### Hardware

Most of the AMP SCZ site teams included EEG co-investigators and labs equipped with EEG systems; thus, we considered using these systems for the overall AMP SCZ project. However, based on concerns about variability across sites in EEG hardware and stimulus presentation software, as well as possible inadvertent changes to the EEG system settings resulting from running unrelated studies, the AMP SCZ EEG team decided instead to lease identical EEG systems across all AMP SCZ sites to minimize these potential sources of EEG variance. The vendor provided identically configured and calibrated BrainProducts actiCHamp+actiCAP 64-channel high impedance EEG systems, ear insert earphones, response buttons, and recording laptop computers. Sites also purchased identical computer displays for visual stimulus presentation.

The EEG vendor also worked with the AMP SCZ EEG team to program and present the EEG paradigms and tasks via a customized stimulus delivery device that was directly integrated with the EEG amplifiers and recording computer. Custom software run on the recording laptop computer activates each step in the entire recording session serially, including 1) initial entry of subject identification number, 2) instructions to the technician to guide participant set-up (electrode cap placement, gel application, impedance checks), 3) instructions for the technician to read to the participant throughout the session, 4) presentation of EEG task runs via direct connection with the stimulus delivery device, alternating between different task runs using a fixed order, and 5) writing EEG data and imbedded event markers to a file using a standardized naming convention.

The technician prompts and participant instructions were translated into all the languages spoken across the AMP SCZ sites, thereby minimizing language differences as a source of cross-site EEG variation. The EEG recording computer and stimulus delivery device were exclusively dedicated to the AMP SCZ EEG recording session, and sites were instructed not to use the computer for any other purpose. Further safeguarding against such uses, the EEG recording computer was “air-gapped” to prevent interactions with the internet and to minimize risks of altered computer function associated with downloaded software or viruses. The EEG file from each completed session was automatically compressed and named, after which it was transferred via a USB thumb drive to a networked computer for upload to the appropriate AMP SCZ hub data upload site (see also Billah et al.^24^ in this issue).

#### Weekly EEG video calls for site initiation and ongoing monitoring

EEG recordings were reviewed during two weekly one-hour remote video calls, one in the morning and one in the late afternoon (Pacific Standard Time), to accommodate the wide range of time zones represented across the sites. These meetings were critical and created an “EEG community” within the AMP SCZ project, comprising EEG technicians, many site EEG investigators, EEG Team leaders from PRESCIENT and ProNET networks, and EEG Team leaders and data analysts from PREDICT-DPACC. While early on the meetings focused on training and certifying sites to initiate EEG recordings, they continued as a weekly forum for reviewing EEG data and providing data quality ratings. Site recording problems and solutions were discussed in such a way that all sites could benefit, iteratively enhancing site expertise and data quality.

#### EEG recording room set up and review

All sites set up their EEG testing areas according to specific implementation guidelines established and reviewed by the EEG Team, with the objective of having appropriate setups that were comparable across sites. Guidelines specified good lab set-up practices including 1) using chairs with straight backs and non-rolling legs placed at a fixed distance (70 cm) from the computer display, 2) reducing ambient electrical noise by minimizing the presence of electrically powered equipment or active electric cords unrelated to AMP SCZ in the recording room or near the participant, 3) setting uniform practices across sites for the EEG recording room, including keeping the room illuminated and ventilated during recordings to minimize artifacts related to participant sleepiness or sweat. Photographs of site set ups were reviewed on weekly EEG video calls, allowing EEG team leaders and site investigators to discuss how to optimize each site’s EEG recording room. Photographs of all site set ups were also made available on the dedicated AMP SCZ EEG website for sites to review. Site-specific set-up challenges or problems were resolved on a case-by-case basis on the weekly EEG video calls, which all site EEG staff were encouraged to attend.

#### Standard operating procedures (SOP) and training materials

In further support of the EEG data core, an SOP manual was written and disseminated to the sites, detailing instructions for EEG system unboxing and set-up, participant preparation and recording, recording cap and electrode clean-up, and data uploads. The EEG SOP document is also available for public download (https://www.ampscz.org/scientists/sops/). Although the EEG Team considered conducting in-person trainings, the costs of sending a trainer to the many international AMP SCZ sites, or of organizing a centralized event, would have been prohibitive. Additionally, the initial planning and set up of AMP SCZ occurred during the COVID-19 pandemic. Accordingly, procedures and resources were created to support remote oversight of the site EEG set-ups and staff training.

Among the steps taken to promote remote training of EEG technicians at each site was the creation of training videos and documents demonstrating EEG system set-up, participant set-up, EEG clean-up, EEG data uploads, one-bucket tests of electrode integrity, and faulty electrode replacement. Flyers were also created in each of the AMP SCZ site languages for distribution to participants to prepare them for their EEG session (including tips like “wash hair but don’t use conditioner or styling gel”). These videos and documents are available to sites on a dedicated website.

#### Site EEG staff certification

To be certified by the EEG Team to collect EEG data, each site’s technician(s) (typically research assistants with no prior EEG recording experience) had to review all training materials and SOPs, record a full EEG session from a lab volunteer, submit the recording to the proper upload location, and then have their processed data reviewed by the EEG Team leaders, typically on the weekly EEG video calls. To be certified, the technician’s submitted EEG recordings had to show evidence of adherence to the EEG SOPs and exhibit acceptable data quality across a variety of quality control parameters. Technicians were asked to submit additional certification recordings if the initial submission did not meet EEG certification standards.

#### Data processing and quality control (QC) monitoring

Site EEG data were uploaded to the appropriate hub site location and participant ID-labeled folder, preferably within 24 h of the session, and the runsheet entries, including text comments about the session, were entered into the appropriate PRESCIENT or ProNET database. A program created by the DPACC automatically retrieved these EEG files and runsheet data daily and copied them to a server managed by the DPACC (see Billah et al.^24^ in this issue). Data files were then automatically submitted to a processing pipeline developed by the DPACC EEG Team, generating quality control (QC) metrics and visualizations, including 1) a channel x time heat map of the entire EEG recording session, 2) plots showing the number of stimuli presented for each task and the participant’s task performance accuracy, 3) a scalp map showing electrode impedances achieved at the completion of participant set up (target: < 25 kOhms) 4) a scalp map showing electrical bridging between electrodes (indicating excessive application of electrode gel), 5) a scalp map of electrical line noise (50 or 60 Hz, depending on the country), 6) ERP waveform averages and multi-channel butterfly plots, 7) event-related time-frequency heat maps, 8) scalp topography maps for each EEG measure, and 9) resting EEG power spectral density plots.

Data visualizations and QC metrics were automatically uploaded to a web-based password-protected EEG QC dashboard custom-designed by a DPACC software engineer and the primary EEG bioengineer/data analyst. Each site was allowed to access and review their own site’s EEG data. Data uploads for each site were officially reviewed on weekly EEG calls, as well as between calls, by the DPACC/ProNET/PRESCIENT EEG Team leaders, who provided a QC rating for each recording session. QC ratings ranged from 4 to 1, as follows: 4 = Excellent, 3 = Good, 2 = Some Usable Data, 1 = Fail. The criteria used to make these ratings are presented in Supplementary Materials.

The QC data reviews and ratings during weekly EEG Team meetings, led by EEG Team leaders, provided all sites with the opportunity to learn from the experiences of other sites, to track down problems such as missing data or incomplete uploads, and in some cases, to bring participants back to repeat a session if data from a recent recording were not usable. Weekly discussions with site EEG technicians and investigators promoted continuous quality improvements, cross-site harmonization, and broad implementation of best practices.

#### EEG recording system and settings

EEG was recorded at all sites using a BrainProducts ActiChamp 64-channel active electrode system. Electrodes were placed into a 64-channel actiCAP electrode cap, with FCz designated as the reference electrode. Electrode gel was introduced under each electrode using plastic syringes, and impedances were reduced to below 25 KOhms when possible, but impedances below 75 KOhms were considered acceptable. Participants were seated in front of a standard 24-inch 1080p LCD computer display (resolution 1920 × 1080) at a distance of 70 cm (from participant’s nasion to display surface), where they viewed visual stimuli or maintained focus on a central fixation cross. Sound stimuli were presented through Etymotic ear insert earphones at an 80 dB sound pressure level (SPL; C scale). Participant EEG set-up was typically completed in 20–40 min. Once participant electrodes were in place and EEG signals appeared appropriate, the recording session commenced with standard instructions for each EEG task, which included practice trials to ensure the participant understood the task. The EEG paradigms were broken up into runs presented in alternating sequence using a fixed order. A session run sheet, showing the sequence of instructions and task runs, was also provided for technicians to document any recording problems or concerns about participant performance.

### EEG paradigms

#### Mismatch negativity/visual oddball task

As is typical in auditory MMN paradigms, participants were told to ignore the presented sounds while they attended to a primary visual task. As previously implemented^55^, we incorporated a VOD task for participants to perform during the MMN paradigm. Timing of visual stimuli was jittered with respect to onsets of auditory stimuli to prevent simultaneous presentation or systematic differences between auditory and visual stimulus onset times, permitting extraction of separate ERPs for the MMN and the VOD tasks from the same continuous EEG recording while avoiding overlap of their ERP signals.

In the MMN paradigm, auditory stimuli consisted of 90% standard tones (633 Hz, 50 ms duration) and 10% pitch+duration “double-deviant” tones (1000 Hz, 100 ms duration) presented in a pseudorandom sequence. Tones were presented with 5 ms rise and fall times and a 500 ms stimulus onset asynchrony (SOA). A total of 3200 tones were presented over 5 separate runs, with each run starting with 20 standards to facilitate participant’s initial formation of a memory trace for the standard and the corresponding expectation that standards would recur.

The 3-stimulus VOD task consisted of a pseudo-random sequence of frequent (80%) standard visual stimuli (small blue circle, diameter subtending 4° of visual angle, white background), infrequent (10%) target stimuli (large blue circle, diameter subtending 8° of visual angle, white background) and infrequent (10%) novel stimuli (variety of fractal pattern square images flanked by white background on both sides). Each stimulus was presented for 500 ms with an average SOA of 2 s (uniformly jittered in 16.67 ms steps from 1.6 to 2.4 s). During inter-stimulus intervals, the screen remained white, and a small black fixation cross appeared at its center. A total of 800 visual stimuli (80 targets, 80 novels, 640 standards) were presented over five separate runs. Participants were instructed to maintain visual focus on the fixation cross and to press a button with the thumb of their preferred hand in response to target stimuli, but not to novel or standard stimuli.

#### Auditory oddball task

In the 3-stimulus AOD task, a pseudo-random series of frequent (80%) standard tones (1200 Hz tone), infrequent (10%) target tones (500 Hz tone) and infrequent (10%) “novel” sounds (variety of sounds, e.g., dog bark, car horn) were presented with an average SOA of 1.25 s (uniformly jittered between 1.1 s and 1.4 s in 25 ms steps). Tones were 50 ms in duration (5 ms rise/fall time). Novel sounds, selected from a corpus developed by Friedman^116^ and from sound libraries publicly available on the internet, ranged between 175 and 250 ms in duration and had an average intensity of 80 dB SPL (C scale). A total of 800 auditory stimuli (80 targets, 80 novels, 640 standards) were presented over four separate runs. As in the VOD task, participants were instructed to press a button in response to target, but not to novel or standard, stimuli.

#### Gamma 40-Hz auditory steady state response paradigm

The 40-Hz ASSR paradigm consisted of 150 click train trials, each 500 ms in duration, with an SOA of 1.5 s. Each click train comprised 1 ms rarefaction clicks presented every 25 ms, yielding a 40-Hz stimulation frequency to drive the ASSR. Participants were instructed to maintain visual focus on a fixation cross on the computer display while passively listening to click trains.

#### Eyes open/eyes closed resting EEG

Resting EEG was recorded for 185 s during which participants were instructed to keep their eyes open while maintaining visual focus on a fixation cross on the computer display. This was followed by another 185 s of EEG recording during which participants were instructed to keep their eyes closed. For both “eyes open” and “eyes closed” EEG runs, participants were told there would be no sounds or images presented, that they should maintain a comfortable position while minimizing movement, and that they should not fall asleep.

The sequence of task instructions, task runs, and duration of each run are presented in Table 1. The total recording time was 57:23 min.

**Table 1:** AMP SCZ EEG session: Task run order and timing.

Order	Task/Paradigm	Run	Duration (min)
1	MMN/VOD Instructions		0:50
2	VOD Task Practice		1:45
3	MMN/VOD	1	5:20
4	AOD Task Instruction		0:38
5	AOD Task Practice		1:45
6	AOD	1	4:15
7	MMN/VOD	2	5:20
8	AOD	2	4:15
9	MMN/VOD	3	5:20
10	AOD	3	4:15
11	MMN/VOD	4	5:20
12	AOD	4	4:15
13	MMN/VOD	5	5:20
14	40-Hz ASSR	1	2:35
15	Resting EEG (eyes open)	1	3:05
16	Resting EEG (eyes closed)	1	3:05
	TOTAL SESSION TIME		57:23

*MMN* mismatch negativity, *VOD* visual oddball task, *AOD* auditory oddball task, *ASSR* auditory steady state response, *EEG* electroencephalography, *min* minutes.

### EEG task data processing and scoring

#### ERP processing pipeline

Continuous EEG data were digitized with a sampling rate of 1000 Hz and bandpass filtered between DC (high-pass) and 280 Hz (low-pass) during acquisition. Offline, data were subsequently downsampled to 250 Hz and high-pass filtered with a 0.2 Hz cutoff using functions from EEGLAB^117^. Outlier channels were identified and interpolated using tools from the PREP pipeline^118^, which was also used to simultaneously obtain a robust estimation of a common average reference. The common average reference used for ERP analyses explicitly excluded left and right mastoid channels, as well as Fp1 and Fp2, as these channels were prone to noise but were excluded from the pre-processing step that interpolated noisy channels, both to preserve the possibility of using uninterpolated linked mastoids as a reference and to preserve eye movement and blink activity in the Fp1 and Fp2 electrodes that are later used by independent component analysis (ICA) to identify ocular artifacts. Thus, our approach followed the PREP recommendation to leave mastoid electrodes out of the iterative interpolations of bad electrodes implemented during derivation of the robust common average reference, but it further omitted FP1 and FP2 from the robust average derivation.

After continuous EEG data were re-referenced to the robust common average reference, they were separated into epochs time-locked to onsets of task stimuli (for MMN deviants and standards: −0.5 s to 0.5 s; for VOD and AOD targets, novels, and standards: −1 s to 2 s; for 40-Hz ASSR click train trials: −0.25 to 0.75 s). Subsequently, a canonical correlation analysis^119^ was used for blind source separation of muscle artifacts from brain activity. Outlier epochs were identified using rejection criteria from the FASTER^120^ EEG processing pipeline. Specifically, epochs ≥ 3 standard deviations from the mean on epoch mean amplitude, mean variance, or mean peak-to-peak voltage, were rejected and excluded from analyses. Subsequently, ICA was run on the EEG epochs, and ICLabel^121^ was used to identify and remove noise components, most notably from blinks and eye movements. The ICA component rejection criterion was a combined probability of ocular, cardiac, muscular, and noise sources totaling greater than 50%. EEG epochs were baseline corrected by subtracting the −100 to 0 ms baseline mean from all data points in the epoch.

ERP waveforms were generated by averaging all available epochs that survived artifact rejection for each stimulus type and scalp electrode, irrespective of participant performance. The decision was made not to exclude trials with inaccurate responses because sites occasionally reported that participant button presses failed to register consistently. It was determined that this was due to partial presses of the response button, which required a full and rapid press to register responses. ERP components derived from all trials vs correct trials were all highly correlated (for QC ≥ 2: r > = 0.986; QC ≥ 3: r > = 0.989; QC = 4: r > 0.992) and showed equivalent test-retest reliability coefficients. Therefore, we decided to use all trials to maximize the number averaged to generate each ERP wave.

#### ERP component scoring

For the MMN paradigm, after deriving ERPs to deviants and standards, deviant-standard difference waves were generated and used to identify the MMN. MMN was identified as the largest negative peak (126 ms) between 100 and 200 ms post-stimulus onset at electrode FCz in the pooled grand average waves from the CON and CHR groups. MMN was then measured as the average amplitude in a ± 40 ms window centered on this peak (86–166 ms) at each electrode. MMN was maximal at FCz and prominent over a cluster of 6 fronto-central electrodes, from which an average MMN amplitude was derived (Fig. 2E and Table 3).

**Fig. 1:**
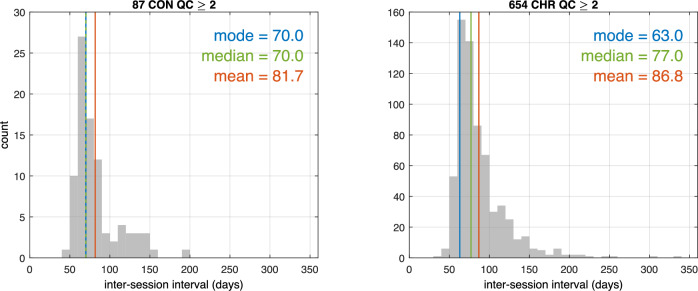
Intervals between baseline and month-2 EEG sessions by group. Frequency distributions of inter-session intervals, in days, between baseline and month-2 EEG sessions, as well as central tendency indicators, are plotted for the full sample (i.e., quality control rating ≥ 2) of community controls (CON) on the left and clinical high risk participants (CHR;) on the right. Mean (orange), median (green), and modal (blue) inter-session intervals (in days) are indicated with colored vertical lines and are presented in the top right corner of each plot. Distributions were skewed to the right, indicating that a minority of participants had longer inter-session intervals. QC quality control.

**Fig. 2:**
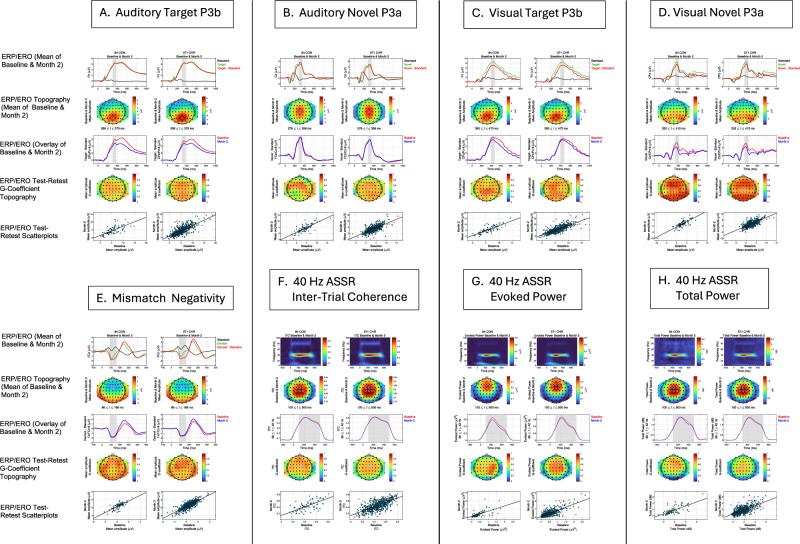
Grand average event-related potential waveforms and event-related oscillation time-frequency maps, scalp topographies, and baseline to month-2 test-retest reliability maps for each task by group. For each event-related potential (ERP; (**A**)-Auditory Target P3b, (**B**)-Auditory Novel P3a, (**C**)-Visual Target P3b, (**D**)-Visual Novel P3a, (**E**)-Mismatch Negativity) or 40-Hz auditory steady state response (ASSR) event-related oscillation (ERO; (**F**)- Inter-trial Coherence; (**G**)-Evoked Power; (**H**)-Total Power) measure, grand average waveforms or time-frequency heat maps, averaged over baseline and month-2 follow-up, from the single scalp electrode (ERP) or fronto-central 6-electrode cluster (ERO) where the measure was most prominent, are shown in row 1. In each case (**A-H**), community control (CON) results are shown on the left, and clinical high risk (CHR) results are shown on the right. For ERP components (**A**–**E**): Row 1 shows grand average ERP waveforms for single stimulus types, as well as difference waves between stimulus types. Light gray vertical bars are centered on the ERP component’s peak and indicate the window over which values were averaged to measure the component’s amplitude. Row 2 shows the scalp topography maps for these ERP component amplitudes, averaged over baseline and month-2 assessments. Row 3 shows baseline and month-2 overlays of the grand average ERP difference waves, averaged over the cluster of electrodes where the component is most prominent (e.g., CPz/Pz-6 auditory target P300 represents average of 6 electrodes centered on CPz and Pz, shown also with white circles around included electrodes in G-coefficient topography maps in row 4). For ERO measures from the 40-Hz ASSR paradigm (**F**–**H**): Row 1 shows time-frequency heat maps for inter-trial coherence (**F**), evoked power (**G**), and total power (**F**). Row 2 shows scalp topography maps for these ERO measures averaged over the 100–500 ms time window and 38–42 Hz frequency band. Row 3 shows baseline and month-2 overlays of the ERO measure's waveform extracted for the 38–42 Hz frequency band. For all ERP and ERO measures (**A**–**H**): Row 4 shows scalp topography maps of the test-retest Generalizability (G)-coefficients, and white circles around the electrodes indicate the electrodes included in the average measure. Row 5 shows scatterplots of these average measures for month-2 vs. baseline.

For the VOD task, after deriving ERPs to standards, targets, and novel stimuli, target-standard and novel-standard difference waves were generated to identify and score target P3b and novel P3a, respectively. Visual P3b amplitude was defined as the average amplitude between 393 and 473 ms, representing a ± 40 ms window centered on the peak latency (433 ms) of target P3b observed at electrode Pz in the pooled grand average waves from the CON and CHR groups. P3b amplitude was maximal over midline electrode Pz and prominent over a cluster of 6 centro-parietal electrodes, which were averaged together to define visual target P3b amplitude. Similarly, visual novelty P3a amplitude was defined as the mean amplitude between 332 and 412 ms at electrode CPz and was averaged over a cluster of 6 centro-parietal electrodes where it was prominent (Fig. 2C, D and Table 3), particularly in the first VOD task run when P3a habituation to novel stimuli was minimal (Fig. 1s, Supplementary Materials).

For the AOD task, ERPs to standards, targets, and novel stimuli were subtracted to generate target-standard and novel-standard difference waves used to identify and score target P3b and novel P3a, respectively. Auditory target P3b peak positivity was identified in the pooled CON and CHR grand average waves at electrode Pz at 339 ms, and it was measured as the mean amplitude between 299 and 379 ms in the target-standard difference waves averaged over the centro-parietal 6 electrodes where P3b was prominent. Similarly, auditory novel P3a was identified as the most positive peak at electrode Cz (316 ms) and measured as the mean amplitude between 276 and 356 ms in the novel-standard difference waves averaged over the fronto-central 6 electrodes where it was prominent (Fig. 2A, B and Table 3), particularly in the first AOD task run when habituation to novel stimuli was minimal (Fig. 1s, Supplementary Materials).

#### 40-Hz ASSR processing pipeline

Time-frequency analysis of EEG single trial data was done with a Morlet wavelet decomposition using FieldTrip software^122^ implemented in MATLAB (http://www.mathworks.com/products/matlab/), as described previously^106,123^. The Morlet wavelet has a Gaussian shape that is defined by a ratio (σ_f_ = f/C) and a wavelet duration (6σ_t_), where f is the center frequency and σ_t_ = 1/(2πσ_f_). Frequencies were calculated in 2-Hz bins for center frequencies from 4- to 100-Hz (i.e., 4-, 6-, 8-,…, 96-, 98-, 100-Hz). The constant (C) was varied over frequencies to optimize the trade-offs between frequency resolution and temporal resolution. For 40-Hz and higher frequencies, C was set to 14. For 20-Hz and lower frequencies, C was set to 7. C was linearly spaced between 7 and 14 for frequencies between 20- and 40-Hz such that spectral bandwidth was equal (6σ_f_ = 17.1429-Hz) across frequency bins in this range. ERP averages were calculated prior to wavelet decomposition to allow calculation of evoked power, but all other time-frequency measures were derived from the single trial data for frequencies between 4- and 100-Hz and timepoints from −248 to 752 ms relative to click-train onset using epochs that spanned −1248 to 1752 ms. For the ASSR paradigm, EEG data were re-referenced to the average of P7 and P8, which are near the mastoids but avoided the noise to which mastoid electrodes were particularly susceptible.

After wavelet decomposition, inter-trial coherence (ITC) was calculated as 1-minus the circular phase angle variance^124^. ITC provides a measure of the phase consistency of frequency specific oscillations with respect to stimulus onset across trials on a millisecond basis. Event-related total and evoked power were calculated by averaging the squared single trial (for total power) or ERP (for evoked power) magnitude values in each 2-Hz frequency bin on a millisecond basis. The average total power values were 10log_10_ transformed and then baseline corrected by subtracting the average of the pre-stimulus baseline (−200 to −100 ms) from each time point, separately for each frequency. Evoked power values were baseline corrected by subtracting the average of the pre-stimulus baseline (−200 to −100 ms). ASSR ITC, total power, and evoked power values were extracted for statistical analysis by averaging the data across a 100–500 ms time window in a 4-Hz bin centered on 40-Hz (38-, 40-, and 42-Hz) from fronto-central electrodes where the signals were most prominent (see Fig. 2F–H and Table 3).

#### Resting-state EEG processing pipeline

The processing pipeline for eyes open and eyes closed resting-state EEG comprised the following steps. Data were high-pass filtered with a 0.5-Hz cutoff, downsampled to 250-Hz, and re-referenced to the mean of all electrodes (common average reference). FASTER EEG automated preprocessing software^120^ was used to identify outlier channels. Continuous EEG data were then divided into 180 1-second epochs. Outlier epochs were determined from the subset of clean channels only. Subsequently, an ICA was run along with ICLabel^121^, and components with greater than 90% probability assigned to ocular, cardiac, or muscular sources were removed. Outlier channels were then interpolated, and a new common “robust” common average was generated and subtracted from individual channels. Power spectral densities (PSDs) were then computed. Finally, absolute EEG power was extracted using conventional EEG frequency band definitions (Delta: 1–3 Hz; Theta: 4–7 Hz; Alpha: 8–12 Hz; Beta: 13–30 Hz; Gamma: 31–48 Hz), averaging over the electrodes where each band was most prominent (see Fig. 4A–E and Table 3).

#### Auditory and visual oddball task performance

**Fig. 3:**
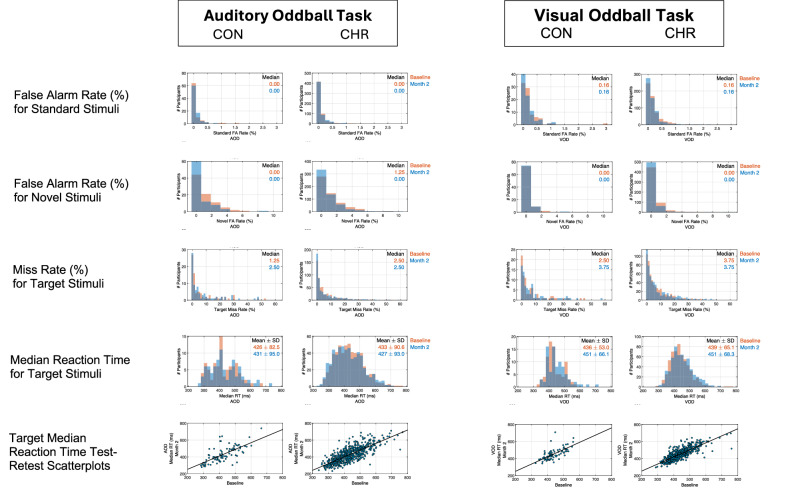
Auditory and visual oddball task performance and target reaction times for baseline and month-2 by group. Performance error rates and median target reaction time distributions at baseline and month-2 follow-up are presented for the auditory oddball task (AOD) on the left and the visual oddball task(VOD) on the right. For each task, community controls (CON) are shown on the left, and clinical high risk participants (CHR) are shown on the right. The distributions of false alarm (FA) rates are shown for standard stimuli (row 1) and for novel non-target stimuli (row 2), and the distributions of miss rates for target stimuli are shown in row 3. Overall, these distributions indicate low false alarm and miss rates, as indicated by the median values shown in the right upper corner of the plots. Row 4 shows the distributions of median target reaction times in milleseconds (ms), and their mean values are presented in the right upper corner of the plots. Row 5 shows the month-2 vs. baseline scatterplots of median target reaction times (RT), corresponding to the G-coefficients presented in Table 3.

For both the visual and auditory oddball task, we calculated error rates for targets (% misses), novel (% false alarms), and standard (% false alarms) (Fig. 3). As noted above, a minority of participants had trouble pressing the response button with sufficient force and vertical angle to register the response consistently, which we expected to spuriously increase target miss rate. Accordingly, target miss rates are presented to demonstrate the relatively minor impact of this problem, as well as to address overall task performance. In addition, for correct target responses, participant median reaction time was calculated (Fig. 3, row 4).

#### Test-retest reliability and stability analyses

Reliability of EEG and behavioral measures over the baseline to 2-month test-retest interval were estimated within CON and CHR groups using Generalizability (G) coefficients^125–127^, a type of intraclass correlation coefficient (ICC)^128^. From a G-theory framework, the study design specified participants (i.e., “Persons’) as the objects of measurement, and test occasion as the single facet of measurement over which reliability, or “generalizability”, of scores was assessed. This Persons x Occasion study design was fully crossed, with Persons and Occasion modeled as random effects, and a restricted maximum likelihood approach (implemented in MATLAB^129^) was used to estimate variance components for Persons (σ_p_), Occasion (σ_o_), and Persons x Occasion (plus Error, σ_po+e_). The G-coefficient error term includes the relative error (σ_po+e_), which reflects changes in the ordering of participants from baseline to month-2 but excludes the contribution to “absolute error” (i.e., σ_o_ + σ_po+e_) from Occasion variance (σ_o_). This is because the main effect of Occasion, if any, applies equally across participants, leaving their relative rank order unaltered from baseline to follow-up. The G-coefficient is calculated using Eq. 1 below:

1 $$G=\,\frac{{\sigma }_{p}^{2}}{\left({\sigma }_{p}^{2}\,+\,\frac{{\sigma }_{{po}+e}^{2}}{{n}_{o}}\right)}$$

where n_o_, the number of occasions over which measurements will be averaged before using them in AMP SCZ, is set to 1. This conforms with our plan to use the baseline assessments alone, or the change between baseline and month-2, as potential predictors of clinical outcomes in CHR individuals. However, if our EEG predictors are averaged over baseline and month-2 assessments, then n_o_ = 2 and the G-coefficients increase relative to those reported here. When n_o_ = 1, the G-coefficient calculated using Eq. 1 is equal to ICC(3,1) defined by Shrout and Fleiss^128^. Adopting previously described guidelines for categorizing ICCs^130^, G-coefficients can be considered poor (G < 0.4), fair (0.4 ≤ G < 0.6), good (0.6 ≤ G < 0.75), or excellent (0.75 ≤ G ≤ 1.0). In addition, to test the mean stability of the measure, the effect of time (month-2 minus baseline) is tested separately within each group using a paired t-test (alpha = 0.05, two-tailed).

G-coefficients presented below are for averages of electrode clusters defined for each measure based on scalp topography. CHR and CON groups comprised participant EEG sessions with QC ratings ≥ 3, as this balances the need to maximize sample size with the need to exclude excessively noisy data. G-coefficients for electrode clusters and for the single electrode where each ERP component is typically maximal, calculated in groups defined by QC ≥ 2, QC ≥ 3, and QC = 4 cut-offs, are presented in Supplementary Materials.

## Results

Participant demographic data are presented in Table 2. Although we refer to EEG assessments as comprising baseline and 2-month follow-up sessions, the modal interval between baseline and “month-2” was somewhat longer, and the within-group distributions were positively skewed (Fig. 1). Among CHR participants, 50% had intervals ≤ 77 days, 75% had intervals ≤ 97 days, 90% had intervals ≤ 125 days, and 98% had intervals ≤ 181 days. Among CON participants, 50% had intervals ≤ 70 days, 75% had intervals ≤ 89 days, 90% had intervals ≤ 125 days, and 98% had intervals ≤ 158 days. In terms of QC ratings, of the EEG sessions rated to date (2,347), 60.8% were rated 4, 33% were rated 3, 5.6% were rated 2, and 0.6% were rated 1. Thus, 93.8% of the sessions were rated as 4 or 3 with only 0.6% of the sessions designated as unusable.

**Table 2:** Group demographics.

Group	n	Sex at Birth		Age (years)	
			Mean	SD	Range
Clinical High Risk	571	222 M (38.9%) 349 F (61.1%)	21.18	4.08	12.42 - 30.92
Community Controls	84	40 M (47.6%) 44 F (52.4%)	21.54	4.08	12.92 - 30.17

Groups are derived from participant sub-sample with EEG session quality control ratings ≥ 3.

*M* male, *F* female, *SD* standard deviation.

### Auditory and visual target P3b

For both AOD and VOD tasks, target ERP waveforms, averaged over baseline and 2-month assessments, showed clear P3b component peaks with typical latencies and a scalp maximum at midline parietal electrode Pz, in both CON and CHR groups, whereas standard stimuli did not evoke a P300 (see Fig. 2A, C, row 1). Target-standard ERP difference waves were used to facilitate isolation of the P3b and its scalp topography (Fig. 2A, C, row 2). Overlay of baseline and 2-month follow-up difference waves, averaged over 6 centro-parietal electrodes (Table 3), showed small but significant declines in P3b amplitude (Fig. 2A, C, row 3) in both CON (AOD: t_83_ = −3.78, p < 0.001; VOD: t_83_ = −3.22, p = 0.002) and CHR (AOD: t_570_ = −8.56, p < 0.001; VOD: t_570_ = −3.45, p < 0.001) groups. Test-retest G-coefficient scalp topography maps (Fig. 2A, C, row 4) and scatterplots for the auditory and visual target P3b electrode cluster (Fig. 2A, C, row 5) show good to excellent G-coefficients in both groups (see Table 3).

**Table 3:** Test-retest reliability for electroencephalography-based and reaction time measures in community control and clinical high risk groups.

Measure	Electrodes included in average	Community Control (n = 84)	Clinical High Risk (n = 571)
		G-coefficient^a^	G-coefficient
Event-related potential measures			
AOD^b^ Target P300 (P3b)	CPz, CP1, CP2, Pz, P1, P2	0.66	0.72
AOD Novel P300 (P3a)	FCz, FC1, FC2, Cz, C1, C2	0.74	0.70
VOD^c^ Target P300 (P3b)	CPz, CP1, CP2, Pz, P1, P2	0.77	0.76
VOD Novel P300 (P3a)	Cz, C1, C2, CPz, CP1, CP2	0.78	0.72
Mismatch Negativity	Fz, F1, F2, FCz, FC1, FC2	0.73	0.78
Event-related oscillation measures			
40-Hz ASSR^d^ Inter-trial Coherence	FCZ, FC1, FC2, Cz, C1, C2	0.68	0.68
40-Hz ASSR Evoked Power	Fz, F1, F2, FCz, FC1, FC2	0.64	0.75
40-Hz ASSR Total Power	FCZ, FC1, FC2, Cz, C1, C2	0.67	0.65
Power spectral density measures			
Resting EEG^e^ (eyes open): Delta	Fz, FCz	0.80	0.77
Resting EEG (eyes closed) Delta	Fz, FCz	0.86	0.79
Resting EEG (eyes open): Theta	Fz, FCz	0.81	0.81
Resting EEG (eyes closed) Theta	Fz, FCz	0.89	0.84
Resting EEG (eyes open): Alpha	Pz, P1, P2, POz, PO3, PO4	0.88	0.85
Resting EEG (eyes closed) Alpha	Pz, P1, P2, POz, PO3, PO4	0.85	0.79
Resting EEG (eyes open): Beta	AF3, AF4, F3, F4, F5, F6, POz, PO3, PO4	0.66	0.62
Resting EEG (eyes closed) Beta	AF3, AF4, F3, F4, F5, F6, POz, PO3, PO4	0.71	0.72
Resting EEG (eyes open): Gamma	AFz, AF3, AF4, Fz, F3, F4, F5, F6	0.54	0.52
Resting EEG (eyes closed) Gamma	AFz, AF3, AF4, Fz, F3, F4, F5, F6	0.44	0.49
Reaction time measures			
AOD Target Median Reaction Time		0.68	0.78
VOD Target Median Reaction Time		0.67	0.79

**Note**. G-coefficients are estimated for P300 and MMN amplitudes, 40-Hz ASSR measures, and for EEG absolute power spectral density for frequency bands Delta (1–3 Hz), Theta (4-7 Hz), Alpha (8-12 Hz), Beta (13-30 Hz), and Gamma (31-48 Hz).

Participant samples are based on EEG sessions with quality control ratings ≥ 3.

^a^G-Generalizability coefficient; an intraclass correlation coefficient reflecting test-retest reliability over baseline and month-2 EEG sessions.

^b^AOD-auditory oddbal task.

^c^VOD-visual oddbal task.

^d^ASSR-auditory steady state response.

^e^EEG-electroencephalography.

### Auditory and visual novel P3a

For AOD and VOD tasks, novel ERP waveforms, averaged over baseline and month-2 assessments, showed typical P3a components with expected peak latencies and scalp distributions for both CON and CHR groups, whereas as noted above, standard stimuli did not evoke a P300 (see Fig. 2B, D, row 1). Novel-standard ERP difference waves facilitated isolation of P3a and its topography for auditory and visual modalities (Fig. 2B, D, row 2). Overlay of baseline and month-2 follow-up difference waves, averaged over 6 fronto-central electrodes for AOD P3a or 6 centro-parietal electrodes for VOD P3a (Table 3), showed small but significant declines for visual P3a amplitude (CON: t_83_ = −5.64, p < 0.001; CHR: t_570_ = −11.94, p < 0.001) but not for auditory P3a amplitude (CON: t_83_ = 0.35, p = 0.97; CHR: t_570_ = −0.80, p = 0.43) (Fig. 2B, D, row 3). Test-retest G-coefficient scalp topography maps (Fig. 2B, D, row 4) and scatterplots for the auditory and visual novel P3a electrode clusters (Fig. 2B, D, row 5) show good to excellent G-coefficients in both groups (see Table 3).

### Auditory and visual oddball task performance and target reaction time

Distributions of AOD and VOD task performance (false alarm rate to standard and novel stimuli, miss rate to target stimuli) at baseline and month-2 follow-up are presented Fig. 3 (rows 1–3). These highly skewed distributions demonstrate that most participants performed these tasks accurately, with very low median target miss rates (< 4%) and even lower median false alarm rates to novel (≤ 1.25%) and standard (≤ 0.16%) stimuli. Median target RT (Fig. 3, row 4) for VOD showed small but significant increases from baseline to month-2 (CON: t_83_ = 2.71, p = 0.008; CHR: t_570_ = 6.31, p < 0.001), whereas for AOD, RT showed no change in CON (t_83_ = 0.66, p = 0.51) and a small reduction in CHR (t_570_ = −2.42, p = 0.016). G-coefficients showed target RTs to have good reliabilities in CON and excellent reliabilities in CHR groups (Fig. 3, row 5; Table 3).

### Mismatch negativity

For MMN, ERP waveforms, averaged over baseline and month-2 follow-up assessments, showed expected fronto-central negativity between 86 and 166 ms following pitch+duration double-deviants, but not following standards, for both CON and CHR groups (see Fig. 2E, row 1). Deviant-standard ERP difference waves facilitated isolation of MMN and its topography (Fig. 2E, row 2). Overlay of baseline and month-2 difference waves, averaged over 6 fronto-central electrodes (Table 3), showed a small decline in MMN amplitude (Fig. 2E, row 3) that was significant in CHR (t_570_ = 5.61, p < 0.001) and trended toward significance in CON (t_83_ = 1.88, p = 0.064). Test-retest G-coefficient scalp topography maps (Fig. 2E, row 4) and scatterplots for the MMN electrode cluster (Fig. 2E, row 5) show good to excellent G-coefficients in both groups (see Table 3).

### 40-Hz ASSR

For the 40-Hz ASSR, time-frequency heat maps, averaged over baseline and month-2 assessments, showed expected EEG gamma ITC, evoked power, and total power responses driven by 40-Hz 500 ms click trains in CON and CHR groups (Fig. 2F–H, row 1). Gamma ITC and total power were maximal over central midline electrodes, whereas evoked power was maximal over fronto-central electrodes (Fig. 2F–H, row 2). Extracted gamma (38–42 Hz) waveforms over time showed a steep initial ramp up of the 40-Hz-driven gamma response during the first 100 ms of the click train, the peak response at 200 ms, a small decline until 500 ms when the click train ended, and a steep decline to baseline by 600 ms. Overlays of baseline and month-2 gamma waveforms, averaged over the 6 central or fronto-central electrodes (Table 3) are shown in Fig. 2F–H (row 3). Gamma ERO measures, averaged between 100-500 ms post-train onset, did not change significantly in either group for ITC (CON: t_83_ = −0.57, p = 0.57; CHR: t_570_ = −1.46, p = 0.15) or total power (CON: t_83_ = −0.88, p = 0.38; CHR: t_570_ = −1.65, p = 0.10). However, evoked power showed a small but significant decline in CHR (t_570_ = −2.42, p = 0.016) but not in CON (t_83_ = −1.57, p = 0.12). Test-retest G-coefficient scalp topography maps (Fig. 2F–H, row 4) and scatterplots for scalp electrode clusters where gamma ERO measures were largest (Fig. 2F–H, row 5), show good to excellent G-coefficients in both groups for ITC, evoked power, and total power (see Table 3).

### Eyes open/eyes closed resting EEG

**Fig. 4:**
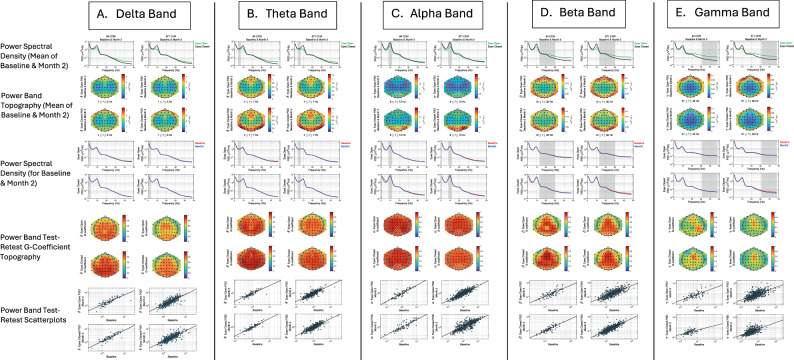
Resting EEG power spectral densities, scalp topographies, and baseline to month-2 test-retest reliability maps by group. Power spectral density (PSD) plots of resting electroencephalography (EEG) for eyes open (green line) and eyes closed (black line) conditions, averaged over baseline and month-2 follow-up, are presented in row 1 for community controls (CON) on the left and clinical high risk (CHR) participants on the right for conventional EEG frequency bands Delta (**A**), Theta (**B**), Alpha (**C**), Beta (**D**), and Gamma (**E**). PSD values are plotted on a log scale. Gray vertical bars indicate the definition of each frequency band. Rows 2 and 3 show the scalp topography maps of PSD for each frequency band (**A**−**E**) during eyes open and closed conditions, averaged over baseline and month-2 follow-up assessments. White circles indicate the electrodes where the PSD was most prominent. PSD plots averaged over the white circled electrodes in the topography maps are overlaid for baseline and month-2 for eyes open (rows 4) and eyes closed (row 5) conditions. Scalp topography maps of test-retest G-coefficients for PSD values at each frequency band (**A**−**E**) are shown for eyes open (row 6) and eyes closed (row 7) conditions. White circles indicate electrodes over which PSDs were averaged, and scatterplots of month-2 vs. baseline PSD values, corresponding to the G-coefficients presented in Table 3, are presented for eyes open condition in row 8 and for eyes closed condition in row 9. For these scatterplots, PSD values are plotted on a log scale.

Resting EEG power spectral density (PSD) plots and frequency band scalp topography maps for eyes open and eyes closed conditions, averaged over baseline and month-2 assessments, are presented in Fig. 4A–E (rows 1-3). PSDs for each band were averaged over scalp electrodes where they were most prominent. Delta (Fig. 4A) and theta (Fig. 4B) PSDs were averaged over frontal midline electrodes (Table 3). Alpha PSD (Fig. 4C), which showed the expected increase in power with eyes closed relative to eyes open (row 1), was averaged over parieto-occipital electrodes (Table 3). Beta PSD (Fig. 4D) was averaged over lateral frontal and midline/lateral parieto-occipital electrodes, and gamma PSD (Fig. 4E) was averaged over frontal electrodes (Table 3). Both gamma (Fig. 4E) and the higher range of beta (Fig. 4D) showed greater PSD during eyes open relative to eyes closed conditions (row 1).

Overlays of baseline and month-2 PSD plots highlighting each frequency band are presented in Fig. 4A–E (rows 4−5). Resting delta PSD, assessed during eyes closed, showed a small significant decline from baseline to follow-up in CHR (t_570_ = −2.20, p = 0.016). No other significant changes in resting EEG frequency band PSDs were evident in either group. Test-retest G-coefficient scalp topography maps and scatterplots for scalp electrode clusters (Fig. 4A–E, rows 6−9) for each resting EEG frequency band showed G-coefficients for each group that were excellent (delta, theta, alpha), good (beta), or fair (gamma) (see Table 3).

### ERP changes over task run and session

**Fig. 5:**
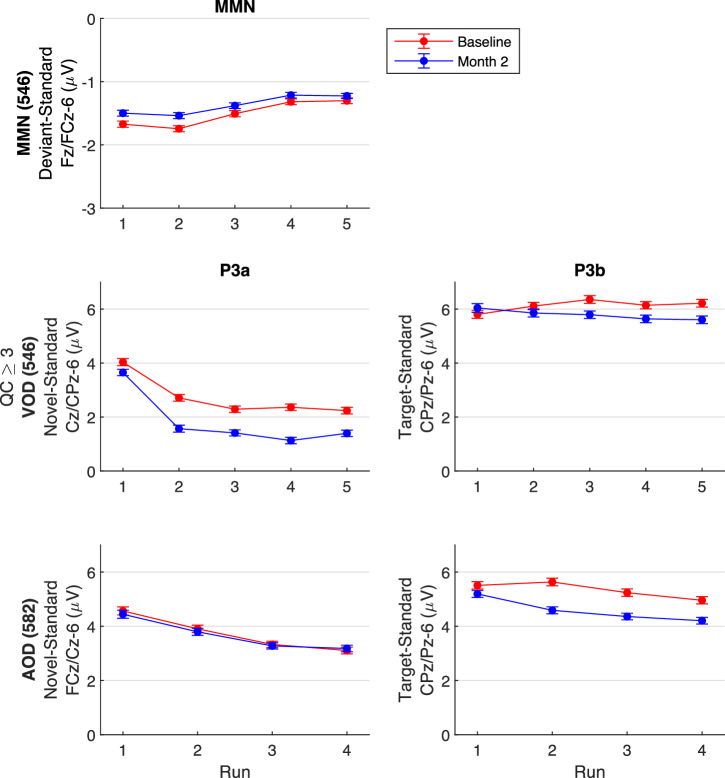
ERP component amplitudes by task run and EEG session. ERP component mean amplitudes and standard error bars are presented by run for baseline and month-2 sessions for mismatch negativity (MMN; N = 546; top row), visual oddball (VOD) novel P3a and target P3b (N = 546; middle row), and auditory oddball (AOD) novel P3a and target P3b (N = 582; bottom row) using clusters of 6 electrodes centered on midline electrodes indicated in each plot (e.g., Fz/FCz-6 cluster comprises mean of electrodes Fz, F1, F2, FCz, FC1, FC2). QC = Quality Control. Included data were for sessions where all runs were completed and where the QC rating for both sessions was ≥ 3.

ERP component amplitudes showed significant declines from baseline to month-2 sessions for visual and auditory target P3b, visual novel P3a, and MMN, but not for auditory novel P3a. To further explore the role of habituation of these ERP component amplitudes over repeat assessments, we examined their changes over task run (1-5 for VOD and MMN, 1-4 for AOD) across all participants with complete run data irrespective of group (AOD: N = 582, including 506 CHR and 76 CON; VOD/MMN: N = 546, including 478 CHR and 68 CON) at baseline and month-2 sessions using 2-way Session x Run repeated measures analysis of variance (ANOVA), with follow-up polynomial trend analyses of both linear and quadratic trends over run. For these analyses, alpha was set to p < 0.05, two-tailed, with Greenhouse-Geisser adjustment for non-sphericity. Mean ERP amplitudes across runs and sessions are plotted in Fig. 5 and run-specific topographic maps for AOD and VOD P300 amplitudes are shown in Supplementary Materials (Fig. 1s). ANOVA results are presented in Table 4. For target P3b, both visual and auditory tasks showed significant Session x Run interactions, with the P3b changing relatively little over runs during baseline but declining over runs at 2 months, which was reflected in significantly different linear and quadratic trends over runs between sessions. Visual novel P3a also showed a significant Session x Run interaction, and follow-up trend analyses showed significantly steeper linear and quadratic trends at month-2 relative to baseline. In contrast, auditory novel P3a and MMN showed significant Run effects that did not significantly interact with Session. For auditory P3a, both linear and quadratic trends significantly described the amplitude decline over runs, whereas for MMN, the linear, but not the quadratic, trend was significant. Run 1 amplitude at baseline and month-2 did not significantly differ for AOD P3a (t_581_ = −0.96, p = 0.34) and VOD P3b (t_545_ = 1.83, p = 0.068), indicating that declines over run at baseline had recovered by the first run of the month-2 session. In contrast, run 1 amplitude was significantly smaller at the month-2 session relative to baseline for AOD P3b (t_581_ = −2.70, p = 0.007), VOD P3a (t_545_= −3.24, p = 0.001), and MMN (t_545_ = 4.25, p < 0.001), indicating less than full recovery of run 1’s baseline amplitude at the month-2 session.

**Table 4:** Session × Run repeated measures ANOVA results for ERP components.

Effect				Visual oddball						Auditory oddball					
	MMN			Target P3b			Novel P3a			Target P3b			Novel P3a		
	F	df	p	F	df	p	F	df	p	F	df	p	F	df	p
Session	32.186	1,545	<0.001	17.70	1,545	<.001	186.216	1,545	<0.001	79.78	1,581	<0.001	0.39	1,581	0.533
Run	65.841	4,2180	<0.001	1.46	4,2180	0.13	372.76	4,2180	<0.001	40.66	3,1743	<.001	118.83	3,1743	<0.001
*-linear*	*183.75*	*1,545*	*<0.001*	*0.22*	*1,545*	*0.64*	*411.42*	*1,545*	*<0.001*	*91.25*	*1,581*	*<0.001*	*233.04*	*1,581*	*<0.001*
*-quadratic*	*1.6*	*1,545*	*0.21*	*2.71*	*1,545*	*0.10*	*267.67*	*1,545*	*<0.001*	*0.06*	*1,581*	*0.813*	*25.3*	*1,581*	*<0.001*
Session x Run	1.63	4,2180	0.17	10.249	4,2180	<0.001	9.22	4,2180	<0.001	9.99	3,1743	<0.001	0.81	3,1743	0.484
*-linear*	*5.29*	*1,545*	*0.02*	*25.51*	*1,545*	*<0.001*	*9.87*	*1,545*	*0.002*	*6.25*	*1,581*	*0.013*	*1.84*	*1,581*	*0.18*
*-quadratic*	*0.18*	*1,545*	*0.67*	*7.44*	*1,545*	*0.007*	*19.814*	*1,545*	*<0.001*	*19.25*	*1,581*	*<0.001*	*0.34*	*1,581*	*0.56*

Main effects and interaction probabilities are Greenhouse-Geisser adjusted for non-sphericity.

*df* degrees of freedom, *p* probability value, *MMN* mismatch negativity.

## Discussion

AMP SCZ is the largest research study of CHR individuals and CON undertaken to date, with harmonized measures and procedures implemented across 43 international sites. Implementation of EEG-based paradigms and measures for this study presented many methodological challenges and required many decisions about what to collect and how best to collect it. In this paper, we describe the scientific and methodological considerations underlying these decisions, which were reached through many meetings and discussions by members of the EEG Team. The results included decisions to use identical EEG acquisition and stimulus presentation hardware and software at all sites, dedicated solely to AMP SCZ EEG data collection. In addition, weekly group meetings to review site EEG room set-ups, development and dissemination of detailed SOP documents, and creation of training videos and site technician certification procedures, were implemented to create a community of EEG data collectors and investigators who regularly met to track the progress of EEG data collection. Working closely with the DPACC, a web-based dashboard was created to support weekly reviews of EEG data QC metrics and visualizations, as well as results depicting single subject ERP/ERO and EEG waveforms and scalp topographies. Data quality criteria were developed to rate each EEG session, both to flag problems and to provide a vehicle for ongoing site training and monitoring. The EEG team converged on paradigms and EEG-based measures. ERP component measures included P300 to target (P3b) and novel (P3a) stimuli in both AOD and VOD tasks, MMN to pitch+duration double-deviant stimuli. ERO measures included gamma ITC, total power, and evoked power acquired during a 40-Hz ASSR paradigm. EEG measures from resting EEG with eyes open and eyes closed included power spectra and PSD measures for delta, theta, alpha, beta, and gamma frequency bands.

Taking advantage of the fact that the AMP SCZ study design called for biomarker assessments at baseline and month-2 follow-up, we were able to calculate test-retest reliability/generalizablity (G) coefficients for the main ERP/ERO/EEG measures of interest using relatively simple extractions of ERP component amplitudes, stimulus-driven power/phase synchrony, and EEG band PSD measures based on time and/or frequency windows from scalp electrodes where activity was most evident. Two months is a relatively long interval over which to assess test-retest reliability because true change, and not just random measurement error, may contribute to observed change over time. Moreover, the median interval between baseline and the month-2 follow-up was between 10 and 11 weeks, and distributions showed a positive skew with many participants having substantially longer intervals, increasing the potential for true change to influence observed change. The variability of these intervals across participants likely contributed to changes in participant rank order at follow-up relative to baseline, thereby increasing the Persons x Occasion interaction variance and estimated measurement error (i.e., ${\sigma }_{{po}+e}^{2}$ in Eq. 1). Accordingly, the G-coefficients reported here can be considered conservative estimates of the true reliability of the measures had shorter and more uniform test-retest intervals been used. G-coefficients for most of the measures indicated at least good reliability, with a number reaching the excellent range. Only resting EEG gamma PSD measures showed reliabilities in the fair range, consistent with the lower signal-to-noise ratio of gamma oscillations relative to the larger magnitude lower frequency oscillations in resting EEG.

In terms of changes observed from baseline to month-2 follow-up within each group, both auditory and visual target P3b and visual novel P3a showed significant amplitude reductions, consistent with prior studies that have documented habituation effects on P300 with repeated task exposure, both across trial blocks within a single EEG test session^131–136^ and across test sessions separated by 7–10 days^137,138^. Curiously, auditory novel P3a amplitude did not show the same reduction from baseline to month-2 despite prior evidence that both auditory and visual P300 elicited in passive oddball paradigms (P3a) are more susceptible to rapid habituation^131,132,139,140^ than the P300 elicited in active paradigms (P3b). This is generally attributed to the greater “signal value” afforded to the infrequent stimulus when it is a target requiring a response, rendering P3b initially resistant to habituation over target repetitions^133,134,141^, with habituation emerging only after presentation of a relatively large number of targets (e.g., 200)^133^. Nonetheless, the best evidence for P300 showing habituation-like decline over sessions separated by intervals of 7–10 days (for at least 1 month) comes from studies of P3b elicited in active tasks^137,138^.

As in prior studies showing P300 habituation over trial blocks or sessions^133,135,136,138^, the current study’s decline in P3b amplitude does not appear to reflect a deterioration in task performance in either group, as oddball task performance was generally quite high at both assessments. Further investigation of P300 amplitude declines over run indicated that at baseline, target P3b showed little decline over run, consistent with minimal habituation effects with repeat target presentations. In contrast, habituation over runs was evident for auditory and visual target P3b at the 2-month follow-up session, suggesting that prior oddball task exposure at baseline led to more rapid habituation over runs at follow-up. This emergent habituation over runs underlies the overall decline in target P3b amplitudes from baseline to month-2. Consistent with prior reports showing novel P3a to be more susceptible to rapid habituation over runs^131,132,139,140^, both auditory and visual novel P3a showed significant habituation-like amplitude declines over run at both sessions. In the case of visual novel stimuli, the decline over runs was steeper at the 2-month follow-up, suggesting that prior exposure to the novel images at baseline led to greater and more rapid habituation over runs at follow-up. The initial amplitude decline of visual novel P3a from run 1 to run 2 within each session was particularly pronounced, with the typical P3a central midline scalp topography evident at run 1 being substantially diminished by run 2 (Fig. 1s, Supplementary Materials). In contrast, auditory novel P3a showed the same prominent habituation-like amplitude declines over runs at both EEG sessions, suggesting that habituation to auditory novel sounds did not intensify at the 2-month follow-up. Furthermore, despite this habituation, the auditory P3a fronto-central topography was evident across runs (Fig. 1s, Supplementary Materials).

Most prior studies interpret P3b habituation as reflecting a decrease in the allocation of attentional resources to the task, either because trial repetition induces learning and practice effects that facilitate a shift from more controlled to more automatic processing^133,134,137,142^, and/or because participants tend to over-allocate attentional resources to the oddball tasks in the initial session but learn over trial runs and the repeat session that fewer attentional resources are actually required to perform the tasks^133–136,138^. Another possible explanation for P3b habituation over EEG sessions is decreasing novelty of the task stimuli^136^, although this is not consistent with the failure to observe steeper habituation of auditory novel P3a amplitudes over runs at follow-up relative to baseline. Yet another possibility for P3b habituation over sessions is decreased arousal^135,136^, although this is more likely to play a role within a session as participants become increasingly bored or tired, but is less likely to account for P3b amplitude decline from baseline to the month-2 follow-up. Less is known about habituation effects on MMN over a period of a few months, as was observed here, but there is evidence that early sensory ERP components do show reductions with stimulus repetition consistent with habituation^132,138^. This was further supported by within-session habituation-like declines of MMN over runs at both baseline and month-2 and a small overall reduction in MMN amplitude at month-2 evident across all runs.

In conclusion, the EEG paradigms developed and deployed in AMP SCZ are yielding expected signals, waveforms, and scalp topographies. Moreover, test-retest reliabilities of the measures extracted are mostly good to excellent, supporting their role in AMP SCZ as predictors of clinical outcomes in CHR, as markers of pathophysiological changes and progression over time, and as potential measures of target engagement in future clinical trials with novel drugs. Reliability sets the upper limit on the validity of biomarkers for any of these purposes, and fortunately, none of the measures assessed yielded low enough reliabilities to make their potential validity a concern. At the same time, we present a very limited set of simply quantified ERP/ERO/EEG measures based on one particular pre-processing pipeline. Thus, our results do not preclude the possibility that other pipelines, and other approaches to measurement, could yield measures with higher reliability or stronger validity. Nonetheless, the current report provides critical information and data to support the derived measures released as part of AMP SCZ data uploads to the NIMH NDA. Moreover, analyses reported in the current paper were constrained by the interim nature of the sample. Future studies, working with the complete AMP SCZ EEG data sample, will examine and model normal age relationships in order to account for substantial developmental changes in neural synchrony and cortical networks from early adolescence to adulthood^143^ that potentially contribute to changes in EEG-based measures. Differences between groups, including CON and CHR groups, and CHR sub-groups defined by differences in clinical trajectories and/or clinical outcomes, will also be examined.

## Supplementary information

AMP SCZ Member List & Affiliations

The Electroencephalography Protocol for the AMP SCZ Consortium- Reliability and Stability of Measures_Supplementary Materials

## Data Availability

The data used in this paper are available via scheduled releases at the NIMH Data Archive (NDA) AMP SCZ Data Repository (https://nda.nih.gov/ampscz).
