# Supplementary material for: The electroencephalography protocol for the Accelerating Medicines Partnership® Schizophrenia Program: Reliability and stability of measures
Source: Schizophrenia (Heidelb). 2025 Jun 6;11(1):85. doi: 10.1038/s41537-025-00622-0 (PMC12144291; doi:10.1038/s41537-025-00622-0)
Supplement: Supplementary file 2 — The Electroencephalography Protocol for the AMP SCZ Consortium- Reliability and Stability of Measures_Supplementary Materials [file 41537_2025_622_MOESM2_ESM.docx]

**Supplementary Materials**

**Data Quality Control Ratings and EEG Measure Reliability**

**EEG Quality Control Ratings.** EEG data uploads for each site were officially reviewed on weekly calls, as well as between calls, by the DPACC/ProNET/PRESCIENT EEG Team leaders, who provided a quality control (QC) rating for each recording session. QC ratings ranged from 4 to 1, with 4 = Excellent, 3 = Good, 2=Some Usable Data, 1=Fail. The criteria used to make these ratings are presented below.

4 = Excellent (AOD/VOD task performance: target hit rate ≥ 85%; < 20% false alarm rate to standards or novels; Bridged electrodes: ≤ 2; Electrode impedances: > 50 electrodes with impedance < 25 kOhms; ≤ 4 electrodes with impedance > 100 kOhms; no electrodes with impedance = infinity; adherence to EEG SOPs).

3 = Good (AOD/VOD task performance: target hit rate 60 - 85%; false alarm rate to standards or novels: 20 - 40%; Bridged electrodes: ≤ 2; Electrode impedances: < 50 electrodes with impedance < 25 kOhms; ≤ 20 electrodes with impedance > 100 kOhms; ≤ 4 electrodes with impedance = infinity; partial adherence to EEG SOPs).

2 = Some Usable Data (Low trial counts from trial artifact rejection or incomplete data collection; AOD/VOD task performance: target hit rate < 60%; false alarm rate to standards or novels > 50%; Bridged electrodes: > 10; Electrode impedances: < 20 electrodes with impedance < 25 kOhms; > 20 electrodes with impedance > 100 kOhms; > 4 electrodes with impedance = infinity; poor adherence to EEG SOPs). Sessions with ratings of 2 were flagged for later review to consider which measures, if any, were usable.

1 = Fail (no usable data due to artifacts, technical problems, or poor task performance).

**Participant Quality Control Sub-groups.** To perform a “validity check” of the QC ratings as reflections of lower or higher measurement error, EEG measure reliability G-coefficients were calculated in participant groups based on more stringent (QC = 4) or more lenient (QC ≥ 2) QC score cut offs. A breakdown of the participant full sample and sub-samples, including sample sizes and demographic data, are presented in Supplementary Table 1s. The QC ≥ 3 participant sessions were presented in the main text. Their demographics are presented again here alongside the QC = 4 and QC ≥ 2 participant groups to facilitate comparisons.

Table 1s. Group demographics by EEG quality control rating sub-groups

|  |  |  | **Age** | **(years)** |  |
| --- | --- | --- | --- | --- | --- |
| **Group** | **n** | **Sex at Birth** | **Mean** | **SD** | **Range** |
|  |  |  | **EEG Quality Control ≥ 2** |  |  |
| Clinical High Risk | 654 | 249 M (38.1%)  405 F (61.9%) | 21.10 | 4.12 | 12.42 - 30.92 |
| Community Controls | 87 | 42 M (48.3%)  45 F (51.7%) | 21.46 | 4.13 | 12.25 - 30.17 |
|  |  |  | **EEG Quality Control ≥ 3** |  |  |
| Clinical High Risk | 571 | 222 M (38.9%)  349 F (61.1%) | 21.18 | 4.08 | 12.42 - 30.92 |
| Community Controls | 84 | 40 M (47.6%)  44 F (52.4%) | 21.54 | 4.08 | 12.92 - 30.17 |
|  |  |  | **EEG Quality Control = 4** |  |  |
| Clinical High Risk | 276 | 116 M (42.0%)  160 F (58.0%) | 21.32 | 4.05 | 12.75 - 30.83 |
| Community Controls | 45 | 23 M (51.1%)  22 F (48.9%) | 22.72 | 3.98 | 13.92 - 30.17 |

**Note.** M = male, F = female; SD = standard deviation; For Baseline and/or Month 2 Follow-up, EEG (electroencephalography) Quality Control ratings: 2 = Some Usable, 3 = Good, 4 = Excellent.

**Test-retest Reliability of EEG Measures Based on QC Cut-offs.** Comparisons of G-coefficients from the sub-sample defined by the QC ≥ 3 cut-off with those based on the more liberal (QC ≥ 2) or stringent (QC = 4) cut-offs provide a “validity check” on our QC ratings. To the extent that QC our ratings accurately reflect EEG measurement error, G-coefficients will be larger as the QC cut-off increases (despite the fact that the sample size decreases). This was generally the case, as shown in Table 2s, but was somewhat less evident in the CON group. In the CON group, the mean G-coefficient across EEG measures was 0.70 for the QC ≥ 2 cut-off , 0.71 for the QC ≥ 3 cut-off, but only .67 for the QC = 4 cutoff. The drop in G-coefficient magnitude when using a QC = 4 cut-off is likely due to the reduction in sample size from n=84 to n=45 and an associated reduction in the participant variance (see Table 1s). In the CHR group, where sample sizes were much larger even for the most stringent QC = 4 cut-off, the mean G-coefficient across EEG measures monotonically increased with QC cut-off (QC ≥ 2, G = 0.71; QC ≥ 3, G = 0.72; QC = 4, G = 0.75). These results support the validity of the QC ratings as reflections of measurement error, and further demonstrate that the QC ≥ 3 cut-off yields EEG measures that, on average, have good test-retest reliability.

**Run Effects for AOD and VOD Targets and Novels at Baseline and 2-month Follow-up.** Figure 1s shows the mean amplitudes for clusters of 6 electrodes (shown with white circles) as well as topography maps by Run for baseline and 2-month follow-up, for combined groups of CHR and CON with sessions containing all runs and rated with a quality control (QC) rating ≥ 3 (VOD N=546; AOD N=582). Time (t) windows represented in topographic maps are 332 ≥ t ≥ 412 ms for VOD novels-standards, 393 ≥ t ≥ 473 ms for VOD targets-standards, 276 ≥ t ≥ 356 ms for AOD novels-standards, and 299 ≥ t ≥ 379 ms for AOD targets-standards. Habituation-like declines in target P3b amplitudes are less evident at baseline and only emerge to a significant degree at the 2-month follow-up. Amplitudes decline across runs for both baseline and 2-month follow-up AOD and VOD novel P3a amplitudes, consistent with rapid habituation over runs. Note in particular that the classic central midline novelty P3a topography is really only evident in the first VOD run, indicating particularly rapid habituation of P3a to visual novel stimuli following Run 1 at each EEG session.
